# Lactoferrin for the Prevention of Post-antibiotic Diarrhoea

**DOI:** 10.3329/jhpn.v29i6.9889

**Published:** 2011-12

**Authors:** Alison M. Laffan, Robin McKenzie, Jennifer Forti, Dawn Conklin, Richard Marcinko, Ruchee Shrestha, Michele Bellantoni, William B. Greenough

**Affiliations:** ^1^Department of Epidemiology, Johns Hopkins University, Baltimore, MD 21224, USA; ^2^Division of Infectious Diseases, Johns Hopkins University, Baltimore, MD 21224, USA; ^3^Division of Geriatric Medicine, Johns Hopkins University, Baltimore, MD 21224, USA

**Keywords:** Antibiotics, *Clostridium difficile*, Colitis, Diarrhoea, Diarrhoea, Drug-induced, Elderly, Lactoferrin

## Abstract

Antibiotic-associated diarrhoea (AAD) is a common cause of morbidity and mortality. Older individuals in long-term care facilities are particularly vulnerable due to multisystem illnesses and the prevailing conditions for nosocomial infections. Lactoferrin, an antimicrobial protein in human breastmilk, was tested to determine whether it would prevent or reduce AAD, including *Clostridium difficile* in tube-fed long-term care patients. Thirty patients were enrolled in a randomized double-blind study, testing eight weeks of human recombinant lactoferrin compared to placebo for the prevention of antibiotic-associated diarrhoea in long-term care patients. Fewer patients in the lactoferrin group experienced diarrhoea compared to controls (p=0.023). Based on the findings, it is concluded that human lactoferrin may reduce post-antibiotic diarrhoea.

## INTRODUCTION

Up to 25% of patients treated with broad-spectrum antibiotics experience diarrhoea (1), and this antibiotic-associated diarrhoea (AAD) can vary from mild nuisance loose or watery stools to severe diarrhoea, including colitis. Patients in long-term care are particularly vulnerable, and AAD is an important cause of disability and death in this population (2). However, *Clostridium difficile,* a known aetiologic agent, explains less than half of these cases. The consequences of AAD can be severe, leading to debility due to volume depletion, enteral protein loss, and a chronic inflammatory state (3). Ironically, AAD is also being treated with antibiotics and may contribute to the growing problem of antibiotic-resistant pathogens (4). To prevent post-antibiotic diarrhoea, we employed a normal human breastmilk protein—lactoferrin—which has both anti-inflammatory and antimicrobial properties (5) and has recently been shown to reduce the duration of diarrhoea in children (6). We tested whether recombinant human lactoferrin grown in rice could prevent post-antibiotic diarrhoea in long-term care residents.

## MATERIALS AND METHODS

Nursing-home residents were randomized to receive, by gastrostomy tube, lactoferrin (5 mg/mL) or placebo via a flush solution. Both product and placebo were dissolved in 600 mL of 0.3% saline solution and were similar in appearance, ensuring that patients and staff be blind to treatment condition. The solution was administered each day for 56 days. Nurses and nursing assistants recorded stool quality on each shift, and stool samples were tested for *C. difficile* at enrollment, day 14, 42, and 56.

We conducted an interim analysis when 50% of the enrollment goal was completed (16 participants), leading to a change in the inclusion criteria and the randomization scheme. Thus, the study was divided into two phases—phase 1 and phase 2. During phase 1, patients initially colonized with *C. difficile* (*C. difficile* antigen present in stool samples) were excluded from participation, leading to 38% (6/16) of the participants being disenrolled immediately. Phase 2 amended the protocol such that the participants be only excluded if they had clinically-confirmed disease due to *C. difficile* (stool samples testing positive for *C. difficile* antigen and toxin A and B). Since all participants who were excluded from the study due to *C. difficile* colonization were from the lactoferrin group (6/6), we altered the randomization scheme in phase 2. Phase 2 randomization was changed to two lactoferrin enrollees for each placebo enrollee to ensure a large sample-size enough for comparison between lactoferrin and control groups.

### Study subjects

Patients at the 41-bed ventilator rehabilitation unit of the Johns Hopkins Geriatric Center beginning a new course of broad-spectrum antibiotics (not including metronidazole, vancomycin, and linezolid) were approached for participation in this study, if they had met the following inclusion criteria: (a) nutrition via an enteral feeding-tube; (b) free from antibiotics for 10 days before entry into the study; (c) not allergic to rice or rice products; and (d) not colonized with *C.  difficile* (phase  1) or no signs or symptoms of clinically-confirmed *C. difficile*-assoc-iated disease (phase 2).

### ***C. difficle*** testing

All stool samples were tested for *C. difficile* antigens using a rapid enzyme immunoassay Techlab (CDIFF-CHEK). Samples testing positive for *C. difficile* antigens were further tested for cytotoxins using a cell culture cytotoxicity assay (7). Samples testing positive for *C. difficile* antigen and toxin A and B were classified as infected with *C. difficile.* Samples that tested positive for *C. difficile* antigen only were classified as colonization.

### Statistical analysis

Diarrhoea was defined as two or more loose stools (conforming to the shape of a container) within a 24-hour period. Each 24-hour period meeting this criterion was classified as a diarrhoea-day. Comparisons between lactoferrin and control groups were made on the mean and the total number of diarrhoea-days. Further, an episode of diarrhoea was defined as consecutive days of diarrhoea, ending after two successive 24-hour periods without diarrhoea. The participants were also compared on time-to-first episode of diarrhoea, or time from enrollment into the study to the first diarrhoea-day. The participants never experiencing diarrhoea were censored at the last day of participation in the study as diarrhoea-free.

Non-parametric tests were used for comparisons between lactoferrin and control groups because of the small sample-sizes and failure to meet normality assumptions for continuous data. Categorical variables were compared using Fisher's exact test, and statistical significance was assessed using the Wilcoxon signed-rank test for continuous parameters. The group differences in time-to-diarrhoea were assessed using the log-rank test.

### Ethical aspects

Informed consent was obtained from the patient or healthcare agent before enrollment. The Institutional Review Board of the Johns Hopkins Medicine approved the study.

## RESULTS

Data relating to stool for 22 participants (13 control and 9 lactoferrin participants) were analyzed. The remaining eight participants were excluded from analysis because they did not complete the study. Each of these participants was exited from the study during the first week of participation, and the large majority (6/8) was exited from the study immediately because of a positive *C. difficile* antigen test results, during phase 1 of the study. Two other participants were disenrolled when they were found to have clinical infections due to *C. difficile*. These infections, noted at day 3 and 8, were classified as existing before enrollment into the study by the principal investigator (WBG).

The table compares the lactoferrin group with the control group at enrollment. No significant differences were found in comparisons between the two groups. However, shorter times since the last antibiotic therapy were observed in the lactoferrin group (p=0.07), and these participants were more likely to be enrolled in the second phase of the study (p=0.07).

Fewer patients (4/9; 44.4%) in the lactoferrin group experienced diarrhoea compared to the control group (12/13; 92.3%; p=0.023). Those treated with lactoferrin were at a significantly reduced risk of experiencing diarrhoea compared to the participants in the control group [odds ratio (OR)=0.07, 95% confidence interval (CI) 0.001-0.97]. Comparisons of the mean number of diarrhoea-days (control: 9.3 vs lactoferrin: 4.0 days, p=0.072) and the percentage of the study days with diarrhoea (control: 17.1% vs lactoferrin: 8.0%, p=0.068) showed a trend towards more diarrhoea in the control group. In addition, time-to-diarrhoea was shorter for the control group compared to the lactoferrin group (median time control: 7 vs lactoferrin: 60+ days, p=0.067). This trend can be seen in the figure, which shows the proportion of the participants free from diarrhoea as a function of time since enrollment into the study for lactoferrin (dashed line) and control (solid line) groups.

During the study period, five participants (2 control and 3 lactoferrin) became infected with *C. difficile* (positive for *C. difficile* antigen and toxins), and of these participants, 2/2 in the control group and 1/3 in the lactoferrin group experienced diarrhoea. The differences between control and lactoferrin groups were not significant (p>0.15 for both).

**Table.** Characteristics of participants at enrollment

Characteristics	Lactoferrin (n=9)		Control (n=13)		p value
	Mean or no.	Range or %	Mean or no.	Range or %	
Age (years)	62.1	23.2-83.9	62.4	34.8-91.1	0.92
Male (no., %)	5	55.6	3	23.1	0.38
Black (no., %)	2	22.2	5	38.5	0.65
Anaemia (no.,%)*	5	55.6	4	30.8	0.24
Time (years) on unit	0.5	0.01-1.9	1.6	0.01-6.9	0.35
Time (years) on tube-feeding	0.85	0.14-5.17	0.64	0.04-2.54	0.57
Time (days) since last antibiotic	29	18-48	48	20-99	0.07
Phase 1 of study (no., %)	1	11.1	7	53.9	0.07
Time (days) in study	50.4	19-61	45.6	25-60	0.25
*C. difficile* antigen+ (no., %)	2	22.2	2	15.4	1.00

The differences between lactoferrin and control groups were tested using Fisher's exact test for proportions and Wilcoxon signed-rank test for continuous parameters.

* Anaemia was defined as use of ferrous sulphate

**Fig.**
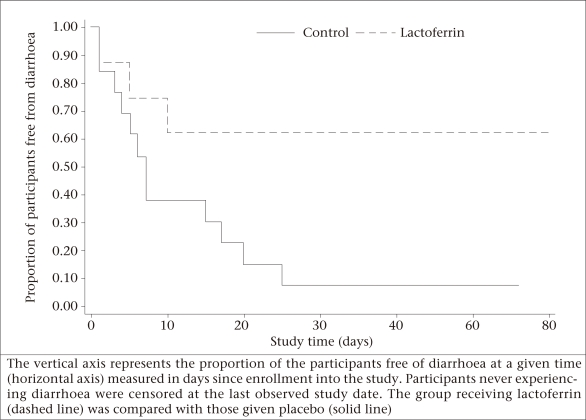
Kaplan-Meier plot of time-to-diarrhoea

## DISCUSSION

Antibiotics destroy normal intestinal microbial flora often leading to diarrhoea (8). Older individuals in long-term care settings are particularly vulnerable (9). The risk of morbidity or mortality due to diarrhoeal disease is higher in those aged over 65 years (2). Although *C. difficile* is recognized as an important cause of AAD, evidence of infection due to *C. difficile* is found in less than half of all cases (3), and current treatment requires administration of antibiotics. Alternative strategies are needed to prevent or treat *C. difficile* and other AADs. The pilot study reported here tested human recombinant lactoferrin as a preventive tool against post-antibiotic diarrhoea.

Lactoferrin, a normal human protein found in breastmilk and leukocytes, has both antibacterial and anti-inflammatory properties (5). This protein does not damage protective microbial flora of the intestine. Human lactoferrin was prepared by inserting the human gene into rice to prepare large amounts at low cost (10) and is nearly identical to native human lactoferrin (11). Administering high concentrations (5 mg/mL) of this product produced no adverse effects in our population of frail older adults, indicating that lactoferrin is safe.

It was recently reported that recombinant lactoferrin with lysozyme reduced the duration of diarrhoea compared to placebo controls in children when delivered via an oral rehydration solution (6). These results suggest that at least some of the effect of breastfeeding to prevent diarrhoea may be due to lactoferrin (12). Here, we have administered this breastmilk component to older individuals; with a result similar to the benefits to infants. Our results offer an interesting alternative approach to preventing and, perhaps, treating post-antibiotic diarrhoea.

Although not the primary outcome, we did monitor for *C. difficile*-associated infection. There were too few infections due to *C. difficile* to assess the effects of lactoferrin on this important cause of post-antibiotic diarrhoea.

In this small study, we had observed a significant reduction in diarrhoea between treatment and placebo groups, suggesting that lactoferrin may be effective in preventing AAD. Caution should be taken in interpreting these results because this study was designed as a pilot project and enrolled a small number of patients. Further, we were forced to alter the study protocol mid-way through the project to achieve balance in treatment groups. However, given our significant findings, this novel preventive approach warrants further investigation in larger trials.

In addition to efficacy trials, more research on the mechanisms through which lactoferrin may work to prevent AAD is needed. Results of research indicate that human lactoferrin provides a readily-absorbable source of iron to any patients with iron-deficiency anaemia (13), and this provides a possible additional benefit of lactoferrin administration. We attempted to investigate this hypothesis post-hoc using information on treatment for anaemia (use of ferrous sulphate) and red blood cell counts; however, due to the small sample-sizes and the post-hoc nature of the analysis, we were unable to show an effect of red blood cell counts in these patients. Future studies should consider the inclusion of a full measure of iron status and parameters of anaemia to explicitly test the potential added benefit of administration of recombinant lactoferrin beyond its antibacterial and anti-inflammatory properties.

Lactoferrin when administered at the start of antibiotic treatment reduced the attack rate of diarrhoea over an eight-week period. There were too few instances of *C. difficile* to assess the effects on this cause of post-antibiotic diarrhoea. Our results offer an interesting alternative approach to preventing and, perhaps, treating post-antibiotic diarrhoea. Further research should incorporate larger sample-sizes powered to determine any effect on *C. difficile* as a serious complication of antibiotic therapy.

## ACKNOWLEDGEMENTS

Ventria Bioscience, the manufacturer of the lactoferrin flush solution, funded this research project. This funding included a small amount of salary support given to Johns Hopkins University for William B. Greenough III, Robin McKenzie, Richard Marcinko, and Alison M. Laffan. Dr. Delia Bethel, a Ventria scientist advised on protocol design, provided the randomization, and assisted with data entry. All analyses were conducted independent of Ventria Bioscience.

The results of this study were presented at the 2009 meeting of the American Geriatric Society, Chicago, IL, USA.
